# Tidal Marshes across a Chesapeake Bay Subestuary Are Not Keeping up with Sea-Level Rise

**DOI:** 10.1371/journal.pone.0159753

**Published:** 2016-07-28

**Authors:** Leah H. Beckett, Andrew H. Baldwin, Michael S. Kearney

**Affiliations:** 1 Marine-Estuarine-Environmental Sciences, University of Maryland, College Park, Maryland, United States of America; 2 Department of Environmental Science and Technology, University of Maryland, College Park, Maryland, United States of America; MESC; University of South Alabama, UNITED STATES

## Abstract

Sea-level rise is a major factor in wetland loss worldwide, and in much of Chesapeake Bay (USA) the rate of sea-level rise is higher than the current global rate of 3.2 mm yr^-1^ due to regional subsidence. Marshes along estuarine salinity gradients differ in vegetation composition, productivity, decomposition pathways, and sediment dynamics, and may exhibit different responses to sea-level rise. Coastal marshes persist by building vertically at rates at or exceeding regional sea-level rise. In one of the first studies to examine elevation dynamics across an estuarine salinity gradient, we installed 15 surface elevation tables (SET) and accretion marker-horizon plots (MH) in tidal freshwater, oligohaline, and brackish marshes across a Chesapeake Bay subestuary. Over the course of four years, wetlands across the subestuary decreased 1.8 ± 2.7 mm yr^-1^ in elevation on average, at least 5 mm yr^-1^ below that needed to keep pace with global sea-level rise. Elevation change rates did not significantly differ among the marshes studied, and ranged from -9.8 ± 6.9 to 4.5 ± 4.3 mm yr^-1^. Surface accretion of deposited mineral and organic matter was uniformly high across the estuary (~9–15 mm yr^-1^), indicating that elevation loss was not due to lack of accretionary input. Position in the estuary and associated salinity regime were not related to elevation change or surface matter accretion. Previous studies have focused on surface elevation change in marshes of uniform salinity (e.g., salt marshes); however, our findings highlight the need for elevation studies in marshes of all salinity regimes and different geomorphic positions, and warn that brackish, oligohaline, and freshwater tidal wetlands may be at similarly high risk of submergence in some estuaries.

## Introduction

Rising sea levels are associated with a host of complications for coastal wetlands, including increases in surface flooding and saltwater intrusion [1]. The ability of coastal marshes (i.e., wetlands dominated by herbaceous plants) to persist under conditions of accelerated sea-level rise is dependent upon maintaining surface elevation relative to sea level [2–5]. Failure to stay abreast of sea-level rise by building vertically results in drowning and conversion of marshland to open mudflats, channel widening, and ponding of marsh interiors [6–7]. Globally, on average sea level rose about 1.7 mm yr yr^-1^ from 1901 to 2010 and accelerated to 3.2 mm yr^-1^ from 1993 to 2010 [8]. Regionally, land subsidence can result in rates of sea-level rise that exceed global averages [9,1]. The 1000-km region along the US Atlantic coast that includes Chesapeake Bay has been identified as a “hot spot” for sea-level rise due to regional land subsidence [10], with an average median rate across seven Chesapeake Bay tide gauges of 4.1 mm yr^-1^ between 1969 and 2014 (3.24–5.11 mm yr^-1^) and accelerating by 0.08–0.22 mm yr^-2^ [11].

To avoid being submerged by rising sea levels, the surface elevation of coastal marshes must increase vertically in the tidal frame at rates that equal or exceed sea level increases [12]. The dynamics of surface elevation change in coastal marshes are controlled by many factors including sea level trends, accretion of mineral and organic matter, decomposition, and vegetation type and productivity [5,12]. Marshes build positively through organic and inorganic inputs including root growth, litterfall, and sediment capture, in addition to temporary increases from soil swelling due to inundation (e.g., because of high tide or increases in sea level) or reduced evapotranspiration [12–14]. Up to a point, sea-level rise can increase marsh elevation by increasing mineral sediment input, reducing decomposition rates, and stimulating plant growth, which further enhances sediment trapping; however, plants will die if sea-level rises too quickly [5,12]. The volume of material contributed by accretion can decrease, and the surface elevation gain dampened, through decomposition of organic inputs as well as export of mineral and organic components, in addition to the condensing effects of autocompaction or dewatering of peat [15–16]. Decomposition rates and export are in part hydrologically controlled, with surface waters carrying away sediment and plant litter, as well as changing the redox state and availability of anions present in sea water that can alter soil respiration rates [15]. Furthermore, excess inundation may reduce plant growth, decreasing organic matter input and reducing root volume [5,17–19].

Processes controlling marsh elevation may vary considerably across the estuarine salinity gradient. Mineral accretionary contributions differ by source and proximity to upland, and thus by position in the estuary [20]. For example, tidal freshwater wetlands have greater amounts of riverine watershed-derived silts and clays whereas salt marshes at the mouths of estuaries have more marine-derived large-grained sand sediments, and are more erodible [21]. Organic accretionary inputs, which are important to marsh vertical accretion, also differ by salinity regime due to differences in plant species composition and productivity. Salinity is a major driver of plant community composition [22], with halophytes contributing different qualities of organic litter (i.e., different amounts of refractory carbon) compared to tidal freshwater communities. Organic matter mineralization rates also differ along estuaries due to differences in amounts of labile organic matter (quantity and quality of organic matter), with more carbon available in tidal freshwater marshes and decreasing amounts down-estuary [23]. Sedimentation may vary along the estuary due to tidal prism dynamics and tidal creek geomorphology. Salt marshes have greater tidal creek sinuosity and more developed banks compared to tidal freshwater marshes, which can impact hydroperiod and hydrodynamics, thus influencing sediment fallout [21]. At the fresh-end of the estuary, tidal flows may be constricted to smaller spaces compared to the mouth of the estuary, which may increase the tidal range and thus influence sediment deposition [21]. Collectively, these studies suggest that deposition rates of organic and mineral matter and decomposition rates vary across the estuarine salinity gradient, which we hypothesize will translate into differences in vertical accretion.

In addition to differences in processes along estuaries, sea-level trends also impact surface elevation. Inter-annual sea-level oscillations have been linked to elevation loss or subsidence. A number of papers (e.g., [24]) have revealed that inter-decadal–and even inter-annual–variability has increased in recent decades, with pronounced short high and low stands from the mean decadal sea levels. Historically, marshes build at rates equal to the rates of sea-level rise [25–26]; however, when sea levels increase too rapidly, marshes cannot accrete sufficiently to maintain positive surface elevation [25, 5]. While the implications of inter-annual high stands for marsh surface elevation change–especially if they are increasing in frequency and magnitude–are evident, pronounced low stands in sea level can drive subsidence rates because of dewatering of peat, decreases in inorganic accretion, and increased surface decomposition [16, 27–28].

In this study, our primary objective was to determine whether marshes of Chesapeake Bay are increasing vertically in elevation at sufficient rates to stay abreast of high rates sea-level rise. Additionally, previous work has focused on surface elevation change in marshes of uniform salinity or estuarine position (e.g., salt marshes, mangroves, tidal freshwater wetlands), and we sought to answer the question, "How do elevation and accretion dynamics vary between marshes of differing salinity?" We hypothesized that due to the variation in accretionary inputs and subsidence across the estuarine landscape, marshes would exhibit differences in the rates of surface elevation change and accretion along an estuarine salinity gradient.

## Materials and Methods

### Site Description

The Nanticoke estuary is a large tributary estuary of Chesapeake Bay. It is microtidal (tidal range of 0.6 m [29]), ebb-dominated [30], and extends from the Chesapeake Bay proper in Maryland into Delaware. It contains tidal marshes of differing geomorphic types, including submerged-upland marshes in the lower estuary and estuarine-meander marshes in the upper estuary. The salinity ranges from about 15 at the mouth to <0.5 in the tidal freshwater zone.

**Fig 1:**
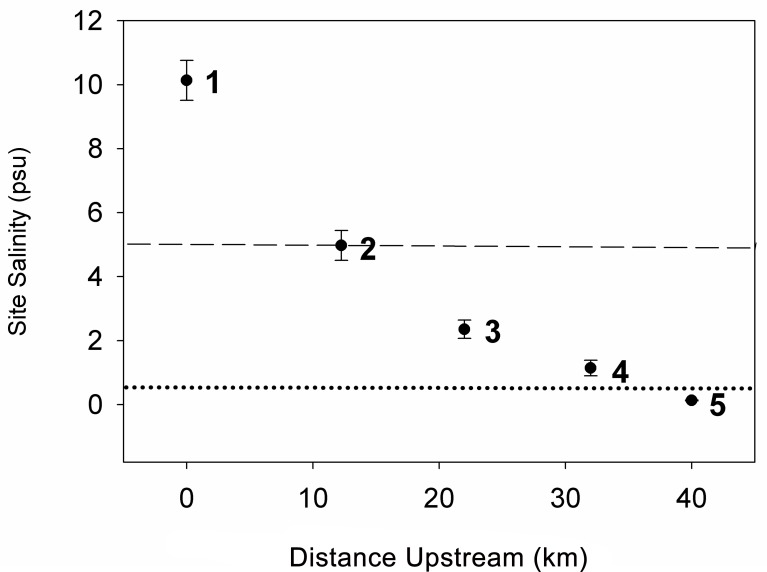
Salinity of study sites on the Nanticoke subestuary. Plotted values are mean ± 1 SE of measurements of marsh surface water and porewater salinity from 2007–2011 (n = 33–39 for each site mean). Porewater measurements were taken at 10–20 cm depths adjacent to surface elevation tables. Distance upstream is distance from Site 1 (0 km upstream). Dashed line represents the oligohaline-mesohaline salinity boundary (5) and dotted line represents the fresh-oligohaline boundary (0.5). Sites are numbered 1–5.

Five sites were established along the salinity gradient of the Nanticoke River. Sites were selected based on salinity zones, and reconnaissance channel salinities taken in interior channels adjacent to marshes during the summer of 2007 were used to space the sites. At each of the five sites, three replicate subsites were established. The sites are 12–14 km apart and subsites are 1–2 km apart. Subsites were chosen to be in interior marsh sections (e.g., at least 30–50 m in from adjacent river or tidal creek channels) because of the reported vulnerability of interior marshes to internal ponding, salt and sulfide accumulation, and decreased sediment deposition [30–32]. Sites were established in similar geomorphic positions (e.g., high marsh, similar distances from tidal creek edge) to minimize possible inter-site differences in hydroperiod, and all sites were vegetated with no evidence of vegetation die-off. The location of subsites within each site was determined randomly. Sites differed in vegetation species composition with upstream tidal freshwater sites dominated by *Peltandra virginica* in the low marsh, and *Bidens* and *Polygonum* spp. in the high marsh. Brackish sites were dominated by *Spartina* spp. and had lower species richness. Salinity was measured using a conductivity-salinity meter (Model 30, YSI Incorporated, Yellow Springs, Ohio). Channel and porewater salinity measurements were taken 2–4 times a year at each subsite for the duration of the study. In total, 9 measurements of channel salinity and 6 measurements of porewater salinity at each SET subsite were made over the course of the study. Measurements were taken at each subsite by placing the probe in the adjacent river or tidal creek at a depth of approximately 30 cm. Porewater measurements were taken within 4 m of each SET by placing the probe in a pilot hole (made with a PVC pipe or stick) at a depth of approximately 10 cm and allowing sufficient water to flow into the hole to cover the conductivity probe (soil in these marshes is continually saturated). Porewater and channel salinity measurements were averaged together at each SET subsite across season and year. Site means were calculated from subsite averages (Fig 1).

Site 1 (38.318462° N, 75.936901° W; datum = WGS84) is a submerged-upland, mesohaline marsh with an average salinity (porewater and channel combined) of 10, and sites 2–5 are estuarine-meander marshes, following the classification of Kearney et al. [6] with average salinities of 5, 2, 1, and 0.1, respectively. Salinity regime of the sites is thus mesohaline (sites 1 and 2), oligohaline (sites 3 and 4), and tidal freshwater (site 5) [33]. Site 1, the most brackish site, was dominated by *Spartina patens* and *Schoenoplectus americanus* in the high marsh, fringed with *Spartina cynosuroides* along the creek banks, as well as a subsite in a *Juncus roemerianus* monoculture. Site 2 (38.40991° N, 75.84022° W), approximately 12 km upstream of site 1, was a *Spartina cynosuroides* marsh with some *Iva frutescens*. Site 3 (38.49857° N, 75.80261° W), 22 km upstream of site 1, had a variety of species including *Spartina cynosuroides*, *Peltandra virginica*, and *Hibiscus moscheutos*. Sites 4 (38.55711° N, 75.7068° W) and 5 (38.61122° N, 75.64228° W) (32 and 42 km upstream of site 1, respectively) were dominated by *Polygonum arifolium*, *Polygonum sagittatum*, *Acorus calamus*, *Symphyotrichum puniceum*, *Typha angustifolia*, *Nuphar lutea*, *Peltandra virginica*, *Sagittaria latifolia*, and *Bidens laevis*. Plant nomenclature follows the United States Department of Agriculture Plants Database (plants.usda.gov, accessed July 29, 2015). Permission was granted by individual landowners to conduct our study in their marshes; permits were not required for observations on state lands.

### Elevation

At each subsite, a Deep-Rod Surface Elevation Table (SET) was installed [34]. The SET is an aluminum arm with 9 fiberglass pins that extend from the arm to the marsh surface. The arm is attached to a fixed elevation point, a receiver installed on stainless steel survey rods that extend from the marsh surface into the underlying Pleistocene substrate (in the case of our SETs, installation depths ranged from 2.4–14.7 m), and surface elevation change is measured as the difference in pin height above the arm over time. The arm rotates around the fixed elevation point (maintaining elevation, but changing the location where pins fall) and locks into place at up to eight possible positions. For each SET, four of the eight positions were used. SETs were installed at each of the 15 subsites in August 2007. Baseline measurements were taken in October 2007, and subsequent measurements were taken twice annually in April and October through 2011. SETs have been used in a variety of coastal habitats [35] such as mangroves, salt marshes, and intertidal zones [16, 28, 36] and are an accurate (± 1.5 mm, [37]) way to collect localized surface elevation change data [34]. It is important to bear in mind that the resulting data from the SET is not absolute elevation change, but instead are measures of the distance from the bottom of the survey rods to the soil surface. Subsidence of deeper strata, as is occurring in the Chesapeake Bay region, will result in a higher rate of sea-level increase than that indicated by SET measurements, meaning that data from this study represent conservative estimates of sea-level increase relative to the wetland surface.

### Accretion

Accretion of mineral and organic matter deposited on the wetland surface was measured using feldspar marker-horizon (MH) plots and cryogenic liquid nitrogen soil cores [37–38]. Powdered feldspar (G200 HP, Feldspar Corporation, Edgar, FL, USA) was laid down and over time accretion was measured as the distance from the top of the white feldspar layer to the surface of the soil using a Vernier caliper. Three marker horizon plots were laid at each SET subsite (3x15 subsites = 45 feldspar plots). Plots were laid when baseline SET measurements were read (October 2007). Subsequently, cores were taken each April and October concurrently with SET elevation measurements. One core at each SET subsite (1x15 subsites = 15 cores per sampling event) was taken per sampling event, and two measurements were read from each core (one on each side of the core) and averaged.

### Data Analyses

Surface elevation change rates were calculated by regressing SET pin height differences (sampling event pin height-baseline height) on day (days since baseline measurements were taken) for each SET pin. Slopes from individual pin regressions were averaged for each SET for subsite surface elevation change rates (elevation change rates are slopes from pin regressions in mm day^-1^, multiplied by 365 for annual rates, mm yr^-1^) [39]. Surface elevation change rates were compared between sites by a one-way analysis of variance (ANOVA) (Proc Mixed procedure using SAS system, version 9.4, SAS Institute, Cary, North Carolina) that compared site means (n = 3 site^-1^). Data were checked to determine whether ANOVA assumptions were met. An estuary-wide average was calculated by averaging surface elevation change rates of the three subsites at each site, then averaging those five site averages. Estuary-wide standard error was calculated using the five site averages. Additionally, to examine the relationship between surface elevation change rates and salinity a linear regression compared subsite salinities with subsite rates of surface elevation change. Site could not be used as a proxy for salinity alone due to the combination of factors independent of salinity that contributed to surface elevation change rates, including proximity to sediment source, for example.

Accretion rates at each subsite (i.e., the rate of surface accumulation of organic and mineral matter) were determined as the slope of linear regression of accreted sediment thickness against day of the study (mm day^-1^, converted to mm year^-1^ for presentation). Accretion rates were averaged by site (e.g., the average of 1A, 1B, 1C for site 1) and an ANOVA was run to examine differences between sites using SAS. Linear regression was used to estimate accretion rate and ANOVA was used to test differences between sites. An estuary-wide accretion rate and standard error were calculated by averaging and taking the standard deviation of the five site means. Linear regression was used to determine whether accretion and salinity were related, as well as whether accretion was a good predictor of surface elevation change.

## Results

**Fig 2:**
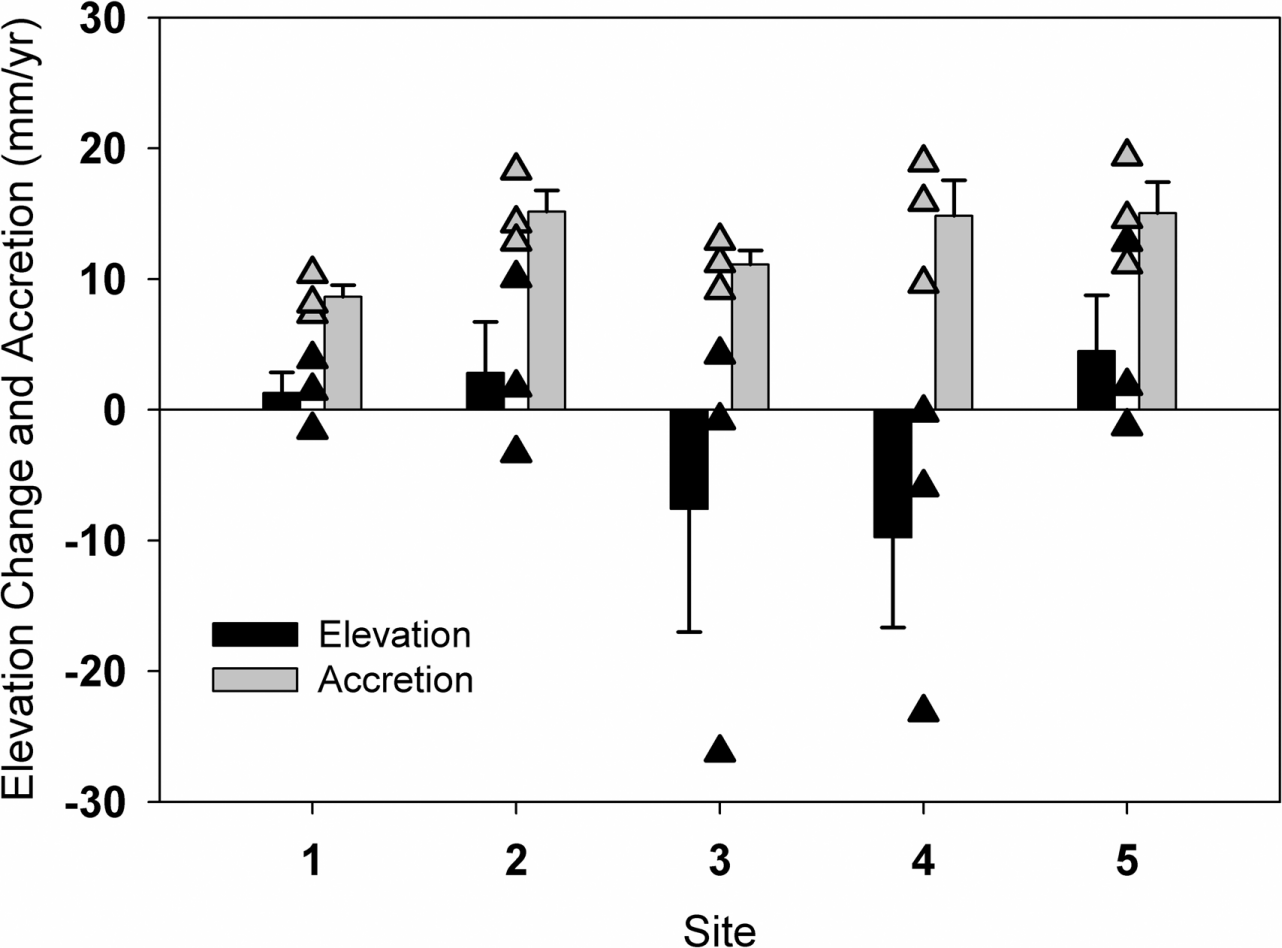
Elevation change (black bars and triangles) and surface accretion (gray bars and triangles) at 5 sites across the Nanticoke River subestuary of Chesapeake Bay. Site numbers 1–5 proceed from downstream brackish marshes to upstream tidal freshwater marshes. Bars represent mean +1 SE of elevation change or accretion rate across 3 replicate subsites, depicted individually as triangles. Rates reflect measurements made from October 2007 through October 2011.

The average rate of elevation change was -1.77 ± 2.7 mm yr^-1^ based on SET measurements across the Nanticoke subestuary over the four-year sampling period. Rates of elevation change did not differ significantly by site (F_4,10_ = 1.22, p = 0.36) (Fig 2), and results of a linear regression between salinity and elevation change reflected very little relationship (R^2^ = 0.03, p = 0.53; results not plotted). The greatest rate of elevation loss among sites was -9.8 ± 6.9 mm yr^-1^ (at site 4, an oligohaline marsh), and the highest increase was 4.5 ± 4.3 mm yr^-1^ (at site 5, a tidal freshwater marsh). Mid-estuarine sites (sites 3 and 4) had high variability within site with elevation change ranges of 30.44 and 22.94 mm yr^-1^, respectively, compared to sites 1, 2, and 5 with ranges of 5.44 mm yr^-1^, 13.37 mm yr^-1^, and 14.10 mm yr^-1^ (subsite rates in S1 Table).

Rates of accretion of matter deposited on the surface were uniformly high across the estuary (~9–15 mm yr^-1^ for site averages, ~7–19 mm yr^-1^at individual subsites; Fig 2, S1 Table), and estuary-wide were 12.95 ± 1.32 mm yr^-1^. Rates of accretion did not significantly differ between sites (F_4,10_ = 2.43, p = 0.12), and ranged from 8.6 ± 0.9 mm yr^-1^ at site 1 (most brackish site) to 15.15 ± 1.63 mm yr^-1^ at site 2 (12 km upstream of site 1). A linear regression reflected a weak and nonsignificant inverse relationship between accretion and salinity (R^2^ = 0.23, p = 0.07). There was not a significant linear relationship between accretion and elevation change either (R^2^ = 0.023, p = 0.46).

## Discussion

Marshes along the Nanticoke River subestuary are on average not keeping pace with sea-level rise. The SET-measured average rate of elevation change was -1.77 ± 2.7 mm yr^-1^ over a four-year period, a conservative estimate of elevation loss given that deeper subsidence processes were not detected by the SET system. Assuming a rate of background sea-level rise of 3.2 mm yr^-1^ [8], this represents an elevation deficit of at least 5 mm yr^-1^. Out of our 15 SET locations, only 4 subsites had rates of elevation increase >3.2 mm yr^-1^. Furthermore, rates of surface elevation change and accretion did not differ significantly among sites along the Nanticoke estuarine salinity gradient, a surprising result given variation in mineral sediment source (fluvial vs. bay), vegetation types, and salinity between tidal marshes of different estuarine position [21]. Surface elevation change and surface matter accretion were not related to measured salinities. Regression analyses examining the relationships between salinity and surface elevation change, salinity and accretion, and accretion and surface elevation change resulted in low R^2^ values that were not statistically significant. Because sea levels will increasingly inundate these marshes to greater depths due to insufficient surface elevation increases, there will likely be negative ecosystem responses such as reduced plant growth and possibly dieback, substrate degradation, and increased erosion across the estuary, ultimately resulting in wetland loss.

The average rates of elevation change and accretion we measured (-9.8 to 4.5 and 8.6 to 15.2 mm yr^-1^) are within ranges reported in other studies (although we report some extremely high rates at individual subsites that are outside the bounds of those reported in the Chesapeake Bay or elsewhere, e.g., elevation change of -26.2 mm yr^-1^ at 3A, accretion maximum of 19.4 mm yr^-1^ at 5B; S1 Table). Delgado et al. [39] examined surface elevation change and accretion in a tidal freshwater marsh of the Patuxent River, another subestuary of Chesapeake Bay, and reported surface elevation change rates ranging from 6 ± 2 mm yr^-1^ to 0 ± 2 mm yr^-1^. A more geographically wide-ranging study [40] compared surface elevation changes in marshes of three estuaries of the U.S. Atlantic coast, including the Patuxent River, where elevation change ranged from -16.2 ± 5.5 to 20.7 ± 16.8 mm yr^-1^ for marshes in lower mesohaline reaches. Outside of the U.S., a study done by Lovelock and others [16] in Moreton Bay, Australia found elevation change rates in salt marshes ranged from 5.9 mm yr^-1^ to 1.9 mm yr^-1^, and accretion rates in salt marshes were 1–3 mm yr^-1^. It is important to note that surface elevation change and accretion rates presented in varying studies may reflect different spatial and temporal scales, and that rates even within an estuary may vary depending on the duration or intervals of measurements.

Though the lack of significant differences among sites may indicate that the contributing processes which differ along the estuarine gradient balanced each other out, we suspect that high intra-site variability (large ranges in rates among subsites) at some locations contributed to the lack of significant difference in rates of surface elevation change. In particular, one SET station each at sites 3 and 4 (both oligohaline marshes), indicated much greater loss than other sites, resulting in large range and SE for those sites. Some of the variability may have been contributed by shrink-swell of marsh soils by hydrologic dilation storage due to evapotranspiraton and tidal flooding, both of which can result mm-scale diurnal changes in elevation in some coastal marshes [35]. Although coordination of measurements at the same point in the tidal cycle might have addressed some of this variability, logistically this was not possible given large distances between sites. Microtopographic differences may also have contributed to high intra-site variability. In these marshes, plant roots often form elevated “hummocks,” possibly as a stress adaptation to reduce soil waterlogging [41], that may increase more in elevation than the lower unvegetated “hollows” between plants due to root growth [42]. Additionally, subsite-specific factors likely contributed to variable intra-site differences such as differences in hydroperiod, quality or source (i.e., marine vs terrestrial) of sediment, and spatial heterogeneity of vegetation communities (e.g., shrub-dominated vs. herbaceous-dominated, monocultures vs. mixtures). These multiple sources of potential variability may suggest that that mechanisms underlying surface elevation change are complex and driven by localized suites of site-specific factors rather than estuary-wide variables. We hypothesize that elevation change may vary considerably within narrow ranges of estuaries; studies using denser networks of SETs, or separating measurements by microtopographic groupings may shed light on this.

### Potential Mechanisms of Rapid Elevation Loss

Though it was surprising our sites along the Nanticoke subestuary salinity gradient did not significantly differ in elevation change or accretion, our findings conclusively demonstrate wetland elevation loss at most of the sites sampled (8 out of 15 lost elevation). Given that elevation loss rates were not significantly related to salinity and estuarine position, and that surface matter accretion was uniformly high across the subestuary, other variables not measured in our study must be driving elevation loss. Here we consider several possibilities.

We suspected that local sea level may have dropped during the course of our study, driving minimal or negative surface elevation change (organic matter oxidation, dewatering, and reduced sedimentation). However, this was not supported: sea level in the Chesapeake Bay rose at 22.8 mm yr^-1^ during the course of our study—much higher than the long-term regional rate of 3.14 mm yr^-1^ [43]. Our study overlapped a 1-in-850 year inter-annual high stand of 128 mm for the U.S. northeast Atlantic Coast that occurred in 2009–2010 [44] and exceeded the decadal average sea level by one standard deviation [45]. Furthermore this high stand occurred within a period showing the highest standard deviation for decadal average sea level (high variability) since the Baltimore tide gauge record began in 1902 [46]. Thus, the high rates of elevation loss we observed in the Nanticoke did not result from sea level fall.

For the Chesapeake Bay region, land subsidence has historically been enhanced locally by groundwater withdrawal due to development or agricultural pressures [47]. It is possible that dewatering of older deeper peats may be linked to lowering the surface elevation of the marsh we studied [48]. Groundwater withdrawal in the Nanticoke watershed is presently high due to the demands of agricultural irrigation [49]. Many agricultural and domestic supply wells in the Delmarva Peninsula are shallow (median depth of 6.7 and 13.7 m, respectively [50], and thus could contribute to shallow aquifer depletion and subsidence in the zone spanned by many of the SETs we used to measure shallow subsidence (< 10m deep).

Because our sites had high rates of surface matter accretion (7–19 mm yr^-1^) but overall elevation loss, we conclude that it was not for lack of mineral and organic matter inputs. Instead, elevation appears to be controlled by subsurface processes. Lovelock and others [16] also concluded that at some their Moreton Bay sites accretion was not a good predictor of surface elevation change, and attributed elevation loss at sites with no accretionary deficit to subsurface processes such as autocompaction, reductions in groundwater, decomposition and root death, and nitrogen pollution. Two important controls on subsurface subsidence are decomposition and plant root growth [12]. Microbial decomposition of organic matter can be accelerated by excess nitrogen. For example, Mendelssohn et al. [51] compared rates of cellulose decomposition (loss in tensile strength of cotton strips) along a salinity gradient and similarly concluded that salinity was not the primary factor driving decomposition, but soil fertility was (concentrations of nitrogen in particular). Given these studies and others [52–58], and the ongoing trend of high nitrogen loading into Chesapeake Bay, we hypothesize that excessive nitrogen may have played a role in driving our negative and low elevation change trends. Further studies of elevation change and accretion should examine porewater and surface nitrogen loading and cycling to determine whether it has an impact on elevation loss and accretion.

## Conclusions

Marshes across the Nanticoke subestuary on average are losing elevation at 1.8 mm yr^-1^, despite surface matter accretion as high as 19 mm yr^-1^, and thus are unable to keep pace with the background rate of sea-level rise of 3.2 mm yr^-1^. Furthermore, rates of accretion and surface elevation change did not differ among sites that spanned tidal freshwater marshes, oligohaline marshes, and brackish marshes, despite differences in vegetation community, salinity, and distance from fluvial and marine sediment sources. To fully understand why accretion and surface elevation change rates did not differ, future investigation should examine the influence of micro-spatial-scale factors such as hydrologic regime, microtopography, decomposition, and primary productivity on elevation change. Estuary-wide follow up studies should examine differences in decomposition rate and litter type, as well as inorganic sediment size and deposition rate of marshes along the estuary. Our findings warn that, in some estuaries, marshes across a range of salinity regimes and geomorphic settings are vulnerable to submergence.

## Supporting Information

S1 Table Subsite locations, elevation change and accretion rates, and salinities measured over four years (2007–2011). (DOC)
