# Supplementary material for: Tidal Marshes across a Chesapeake Bay Subestuary Are Not Keeping up with Sea-Level Rise
Source: PLoS One. 2016 Jul 28;11(7):e0159753. doi: 10.1371/journal.pone.0159753 (PMC4965100; doi:10.1371/journal.pone.0159753)
Supplement: S1 Table — (DOC) [file pone.0159753.s001.doc]

S1

Table: Subsite locations, elevation change and accretion rates, and salinities measured over four years (2007-2011)

| Site | Subsite | Location (Lat and Long) | | Salinity (psu) | Surface Elevation (mm yr^-1^) | Accretion (mm yr^-1^) |
| --- | --- | --- | --- | --- | --- | --- |
| 1 | 1A | 38.3135 | -75.9352 | 9.95 | 3.90 | 7.35 |
|  | 1B | 38.31846 | -75.936901 | 10.55 | 1.50 | 10.41 |
|  | 1C | 38.31758 | -75.940469 | 9.92 | -1.54 | 8.15 |
| 2 | 2A | 38.40379 | -75.83629 | 5.28 | -3.30 | 14.27 |
|  | 2B | 38.40991 | -75.84022 | 5.49 | 10.08 | 18.32 |
|  | 2C | 38.40397 | -75.8424 | 4.25 | 1.71 | 12.87 |
| 3 | 3A | 38.49608 | -75.80388 | 2.25 | -26.20 | 9.18 |
|  | 3B | 38.49857 | -75.80261 | 2.45 | -1.08 | 11.22 |
|  | 3C | 38.49603 | -75.80698 | 2.39 | 4.24 | 12.93 |
| 4 | 4A | 38.55793 | -75.70613 | 1.25 | -0.19 | 18.94 |
|  | 4B | 38.55711 | -75.7068 | 1.42 | -23.14 | 15.91 |
|  | 4C | 38.54929 | -75.717 | 0.82 | -5.93 | 9.66 |
| 5 | 5A | 38.6124 | -75.64175 | 0.14 | 12.85 | 14.61 |
|  | 5B | 38.61122 | -75.64228 | 0.14 | 1.84 | 19.38 |
|  | 5C | 38.60271 | -75.65287 | 0.13 | -1.25 | 11.13 |
